# Real-world efficacy and safety of combined first-line treatment with PARP inhibitors and novel hormonal therapy in mCRPC patients with HRR gene mutations

**DOI:** 10.3389/fgene.2024.1505163

**Published:** 2024-12-06

**Authors:** Andong Guo, Chenrui Wu, Jishuang Cao, Kejia Zhu, Sentai Ding

**Affiliations:** ^1^ Department of Urology, Shandong Provincial Hospital Affiliated to Shandong First Medical University, Jinan, Shandong, China; ^2^ Department of Urology, Shandong Provincial Hospital, Cheeloo College of Medicine, Shandong University, Jinan, Shandong, China

**Keywords:** mCRPC, HRR mutations, PARP inhibitors, NHT, real-world

## Abstract

**Objective:**

This study evaluated the real-world efficacy and safety of combining PARP inhibitors with novel hormonal therapy (NHT) as a first-line treatment in Chinese patients with metastatic castration-resistant prostate cancer (mCRPC) harboring homologous recombination repair (HRR) gene mutations.

**Methods:**

We enrolled 41 mCRPC patients who received at least 1 month of combined treatment with PARP inhibitors and NHT. Patients were divided into two groups: Cohort A (mutations in BRCA1, BRCA2, or ATM genes) and Cohort B (mutations in other HRR genes). The primary endpoint was imaging-based progression-free survival (PFS), with secondary endpoints including objective response rate (ORR), disease control rate (DCR), overall survival (OS), PSA50 response, and adverse events (AEs). To ensure accurate research results and control confounding factors, we will employ multivariate Cox proportional hazards models to evaluate key variables affecting mCRPC patient survival outcomes.

**Results:**

This study enrolled 41 patients, 22 in Cohort A and 19 in Cohort B. The median PFS for all patients was 21.8 months, and the median OS had yet to be reached. The overall ORR was 48.8%, and the DCR was 61.0%. Specifically, the median PFS for Cohort A was 21.8 months compared to 14.5 months for Cohort B. The median OS had yet to be reached for either cohort. Regarding efficacy, 81.8% of patients in Cohort A and 73.7% in Cohort B achieved a PSA50 response. Imaging assessments showed ORRs of 54.6% for Cohort A and 42.1% for Cohort B, with DCRs of 72.7% and 47.4%, respectively. 85.4% of patients experienced grade 1 or 2 adverse events, and 51.2% encountered grade 3 or 4. In the multivariate Cox regression analysis focusing on PFS, the Gleason score was identified as a significant predictor (HR = 5.8, 95% CI: 1.65–20.2, *p* = 0.006).

**Conclusion:**

Combined first-line treatment with PARP inhibitors and NHT is effective and well-tolerated in mCRPC patients with HRR gene mutations, particularly those with BRCA1, BRCA2, or ATM mutations. These findings underscore the potential of this therapeutic combination in managing mCRPC in the Chinese population, suggesting a favorable outcome for those with specific genetic backgrounds.

## Introduction

Prostate cancer is globally the fourth most common malignancy and the eighth leading cause of cancer-related deaths, as reported by GLOBOCAN 2022 (Bray et al., 2024). Initially, most cases are localized and amenable to specific therapeutic interventions. Nonetheless, approximately 30%–40% of patients will experience biochemical recurrence or develop metastatic disease (Henríquez et al., 2021). Furthermore, between 5% and 19% of individuals are diagnosed with *de novo* metastatic prostate cancer, known as metastatic castration-sensitive prostate cancer (mCSPC), a diagnosis that is becoming increasingly frequent (Luyendijk et al., 2023). Despite the use of available treatments, such as traditional chemotherapy and second-generation anti-androgen therapies (Desai et al., 2021), the majority of mCSPC cases will inevitably progress to metastatic castration-resistant prostate cancer (mCRPC), a condition associated with a grim long-term prognosis.

Homologous recombination repair (HRR) is an essential DNA damage repair mechanism for genomic stability. In advanced prostate cancer, notably in mCRPC, mutations in HRR genes occur frequently, with germline and somatic mutations affecting approximately 12% and 20%–25% of patients, respectively (Casadei et al., 2024). BRCA2 is the most commonly mutated HRR gene in mCRPC, found in 44% of cases, followed by ATM, CHEK2, and BRCA1, which comprise 13%, 12%, and 7% of these mutations (Robinson et al., 2015). Prostate cancer in individuals with germline HRR mutations often manifests at an earlier age, displays a more aggressive phenotype, has a higher risk of post-surgical recurrence, and is associated with significantly reduced survival rates compared to those without these mutations. Additionally, these mutations can alter the efficacy of specific treatment (Castro et al., 2013; Page et al., 2019; Tryggvadóttir et al., 2007). Given the substantial prevalence of gene alterations involved in DNA damage repair (DDR), especially within the HRR pathway, there exists a compelling scientific rationale for the application of poly(ADP-ribose) polymerase inhibitors (PARPi) in the management of prostate cancer (Teyssonneau et al., 2021).

Research on the therapeutic use of PARP inhibitors began in the 1970s, targeting a range of disorders, including tumors, stroke, cardiac ischemia, inflammation, and diabetes (Lord and Ashworth, 2017; Slade, 2020). These inhibitors augment the efficacy of radiotherapy and chemotherapy by selectively targeting cells deficient in homologous recombination, particularly those with BRCA1/2 mutations (Gupte et al., 2022; Luo and Keyomarsi, 2022). The primary effectiveness of PARP inhibitors lies in their ability to suppress PARP activity, which is crucial for the repair of single-strand breaks (SSB) in DNA. This suppression results in double-strand breaks (DSBs) accumulation during DNA replication, typically repaired via the BRCA-mediated homologous recombination (HR) pathway. This repair mechanism is disrupted in cells with BRCA mutations, leading to synthetic lethality when combined with PARP inhibitors (Chang et al., 2017; Xavier et al., 2021; Farmer et al., 2005; Bryant et al., 2005).

While randomized clinical trials confirm the efficacy of PARP inhibitors, real-world data are crucial to understanding their impact on a broad and diverse patient population. This is particularly important because participants in clinical trials often have fewer comorbid conditions and demonstrate higher treatment adherence. This study aims to systematically evaluate the real-world efficacy and safety of combined first-line treatment with PARP inhibitors and novel hormonal therapy (NHT) as a primary treatment for mCRPC, focusing specifically on patients with mutations in HRR genes, particularly BRCA1/2 and ATM. Through this approach, the study seeks to bridge gaps in the existing literature and provide actionable insights for clinical practice.

## Materials and methods

### Patients and samples

This retrospective observational study examined real-world data from patients with mCRPC who received combined first-line therapy with PARP inhibitors and NHT at Shandong Provincial Hospital, affiliated with Shandong First Medical University, from 1 January 2022, to 31 October 2024. Eligibility criteria included patients aged at least 18 years with histologically or cytologically confirmed prostate adenocarcinoma featuring at least one documented metastatic lesion identifiable via bone scan, CT, or MRI. Additionally, participants were required to have an Eastern Cooperative Oncology Group (ECOG) performance status of 0–1 and a life expectancy of at least 6 months. Exclusion criteria barred prior systemic treatments in the first-line mCRPC setting, except for androgen depletion therapy. The patients that were included exhibited documented mutations in HRR genes, such as BRCA1, BRCA2, and ATM, as verified by genetic testing before initiating therapy. Patients were divided into two groups based on specific HRR gene mutations, chosen for their prognostic and therapeutic importance in mCRPC. Cohort A included patients with BRCA1, BRCA2, and ATM mutations, which enhance sensitivity to PARP inhibitors due to their key roles in DNA repair (Phan et al., 2023). These mutations typically result in a more aggressive disease but potentially better responses to PARP inhibitors (Sahu et al., 2024). Cohort B consisted of patients with other HRR mutations, which are less well-understood but show variable responses to PARP inhibitors and hormonal therapies (Lukashchuk et al., 2023). This division facilitates a targeted analysis of treatment outcomes, aiming to improve personalized treatment approaches. Diagnoses adhered to the 2016 World Health Organization urological pathology and genetics standards. We meticulously collected demographic and baseline characteristics from the hospital’s electronic medical records system, such as age at diagnosis, Gleason score, tumor stage, treatment history, imaging results, and PSA levels.

### Treatment plan

The treatment regimen included twice-daily doses of 300 mg Olaparib tablets or 60 mg Pamiparib capsules, combined with novel hormonal therapies such as abiraterone and enzalutamide. Monthly patient evaluations included symptom reviews and laboratory tests (prostate-specific antigen (PSA), complete blood count, liver and kidney function, electrolytes, and routine urine and stool tests). Treatment persisted until disease progression, intolerable side effects, death, or consent withdrawal. Efficacy evaluations occurred every four cycles, adhering to RECIST 1.1 and the Common Terminology Criteria for Adverse Events (AEs). The primary endpoint was imaging-based progression-free survival (PFS). Secondary endpoints included the confirmed objective response rate (ORR), defined as the proportion of patients achieving either a complete response (CR) or partial response (PR) on imaging, disease control rate (DCR), the DCR is the sum of the percentages of patients who achieve PR, CR, and Stable Disease (SD), OS, and a PSA50 response, indicated by a reduction of at least 50% in PSA levels. The study also assessed adverse event profiles and additional parameters related to PSA response. Side effect management followed CTCAE v5.0 guidelines. Ethical approval was obtained from the institutional review board, and all participants provided informed consent.

### Statistical analysis

Patient demographics, tumor characteristics, and treatment details were summarized using frequencies and percentages for categorical variables, and interquartile ranges for continuous variables. Kaplan-Meier curves were used for survival analysis. To confirm the accuracy of our findings and control for potential confounding factors, we will employ multivariate Cox proportional hazards models that relate to PFS. These models will incorporate adjustments for several critical variables, including age, Gleason score, baseline PSA levels, and combination therapy, all of which are recognized as influencing survival outcomes in mCRPC patients. By making these adjustments, we aim to provide a more accurate assessment of the effectiveness of combining PARP inhibitors with new hormonal therapies. A p-value of <0.05 was considered statistically significant. All analyses were conducted using SPSS version 27.0.

## Results

### Patient characteristics

**FIGURE 1:**
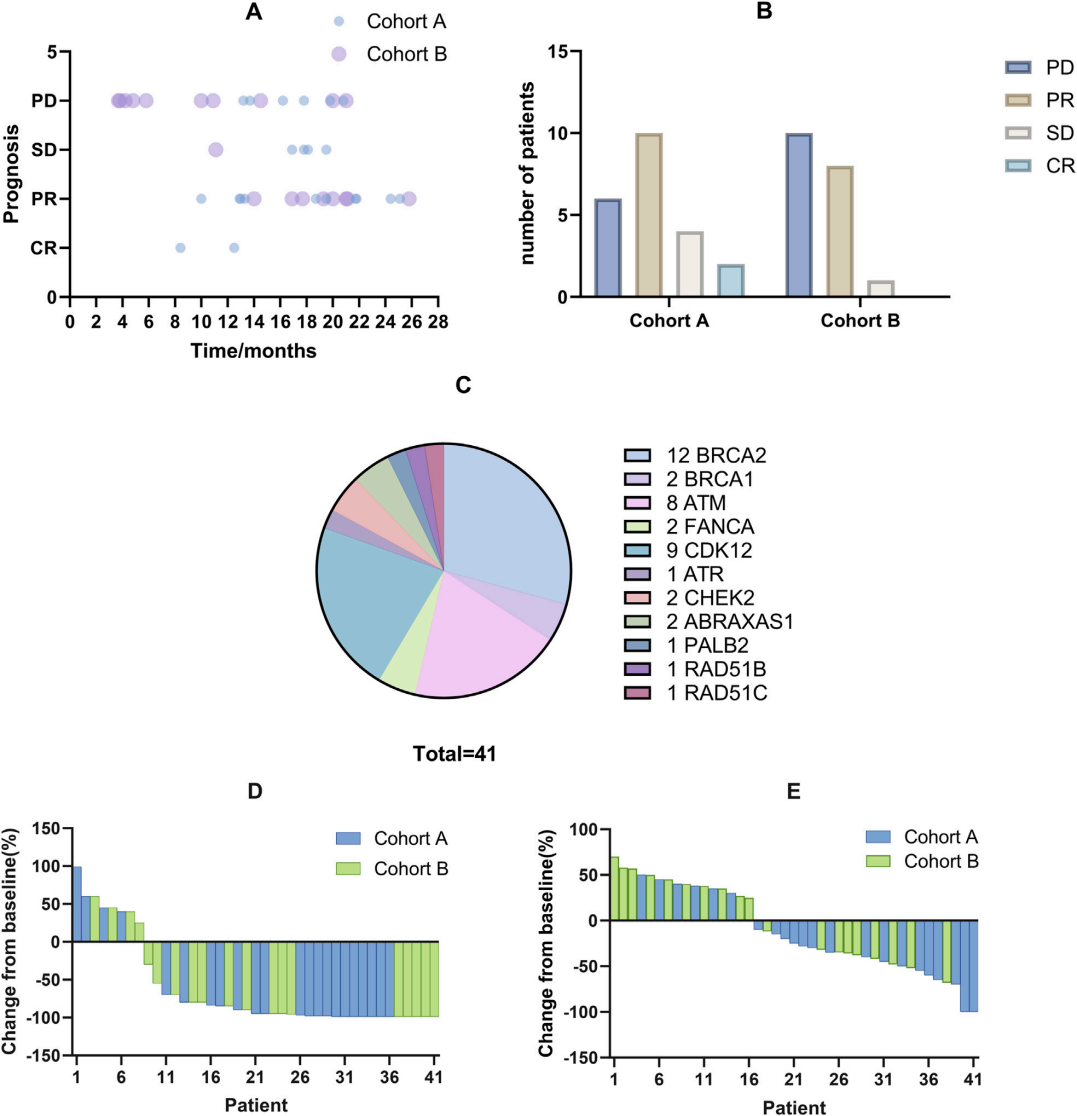
The efficacy of combined treatment with PARP inhibitors and NHT on treating mCRPC patients with HRR gene mutations. **(A)** Each bubble represents an individual patient, detailing the duration of treatment with PARP inhibitors and subsequent prognostic outcomes. Darker bubbles indicate overlapping data points from multiple patients. **(B)** Efficacy evaluation of two cohorts. **(C)** Distribution of HRR gene mutations among patients, depicted in a pie chart. BRCA2 somatic mutations are the most frequent, followed by CDK12 mutations. **(D)** Waterfall plot showing changes in PSA levels from baseline for each patient, indicating individual responses to the treatment. **(E)** Waterfall plot displaying changes in tumor size from baseline for each patient, highlighting the treatment’s effect on tumor reduction.

Table 1 outlines the baseline characteristics of the 41 patients enrolled, stratified before treatment into two groups: Cohort A, comprising 22 patients with at least one mutation in the BRCA1, BRCA2, or ATM genes, and Cohort B, consisting of 19 patients with mutations in other homologous HRR genes. Stratification criteria included ECOG performance status, Gleason score, presence of visceral metastases, and history of systemic therapy. The median age was 70 years (range: 58–83), with the majority having an ECOG performance status of 0 (29.3%) or 1 (70.7%). Notably, 87.8% of the cohort had bone metastases, and over half of both groups had a Gleason score of 8 or higher. All patients received combined NHT. All patients had documented mutations in HRR genes, the most common being BRCA2 mutations (n = 12) and CDK12 mutations (n = 9), as shown in Figure 1. These baseline characteristics demonstrate a well-matched patient population across treatment groups, supporting the validity of the subsequent efficacy and safety analyses. Detailed genetic distributions and additional stratification data are available in the Supplementary Materials.

**TABLE 1:** Baseline characteristics of patients.

Characteristic	Overall (n = 41)	Cohort A (n = 22)	Cohort B (n = 19)
Age			
Median	70	71	68
Range	58–83	58–82	61–83
ECOG score			
0	12 (29.3%)	7 (31.8%)	5 (26.3%)
1	29 (70.7%)	15 (68.2%)	14 (73.7%)
PSA at baseline, ng/mL (IQR)	15.5 (9.6–27.0)	15.5 (9.6–27.0)	10.4 (5.9–22.2)
Gleason score, n (%)			
6–7	17 (41.5%)	11 (50.0%)	6 (31.6%)
8–10	24 (58.5%)	11 (50.0%)	13 (68.4%)
Metastatic site, n (%)			
Bone metastases	36 (87.8%)	19 (86.4%)	17 (89.5%)
Liver metastases	2 (4.9%)	1 (4.5%)	1 (5.3%)
Lung metastases	8 (19.5%)	3 (13.6%)	5 (26.3%)
Lymph node metastases	22 (53.7%)	13 (59.1%)	9 (47.4%)
Combination Therapy, n (%)			
Enzalutamide	7 (17.1%)	4 (18.2%)	3 (15.8%)
Abiraterone	34 (82.9%)	18 (81.8%)	16 (84.2%)

IQR: interquartile range; ECOG: eastern cooperative oncology group; PSA: Prostate-specific antigen; GnRH: Gonadotropin-releasing hormone.

In the multivariate Cox regression analysis focusing on PFS, the Gleason score was identified as a significant predictor (HR = 5.8, 95% CI: 1.65–20.2, p = 0.006). In contrast, age, combination therapy, and baseline PSA levels were not significantly associated with PFS (all p > 0.05). These results underscore the importance of the Gleason score in predicting patient outcomes and guiding treatment decisions. For further details, see Table 2.

**TABLE 2:** Multivariate Cox regression analysis of factors associated with progression-free survival in patients.

Factors	B	Wald	HR (95% CI)	p-value
Age	0.03	2.95	0.93 (0.88–1.09)	0.084
Combination therapy	0.65	1.85	2.45 (0.68–8.85)	0.195
PSA at baseline	0.52	0.92	1.68 (0.49–5.79)	0.396
Gleason score	1.50	7.20	5.80 (1.65–20.20)	0.006

### Effectiveness

**FIGURE 2:**
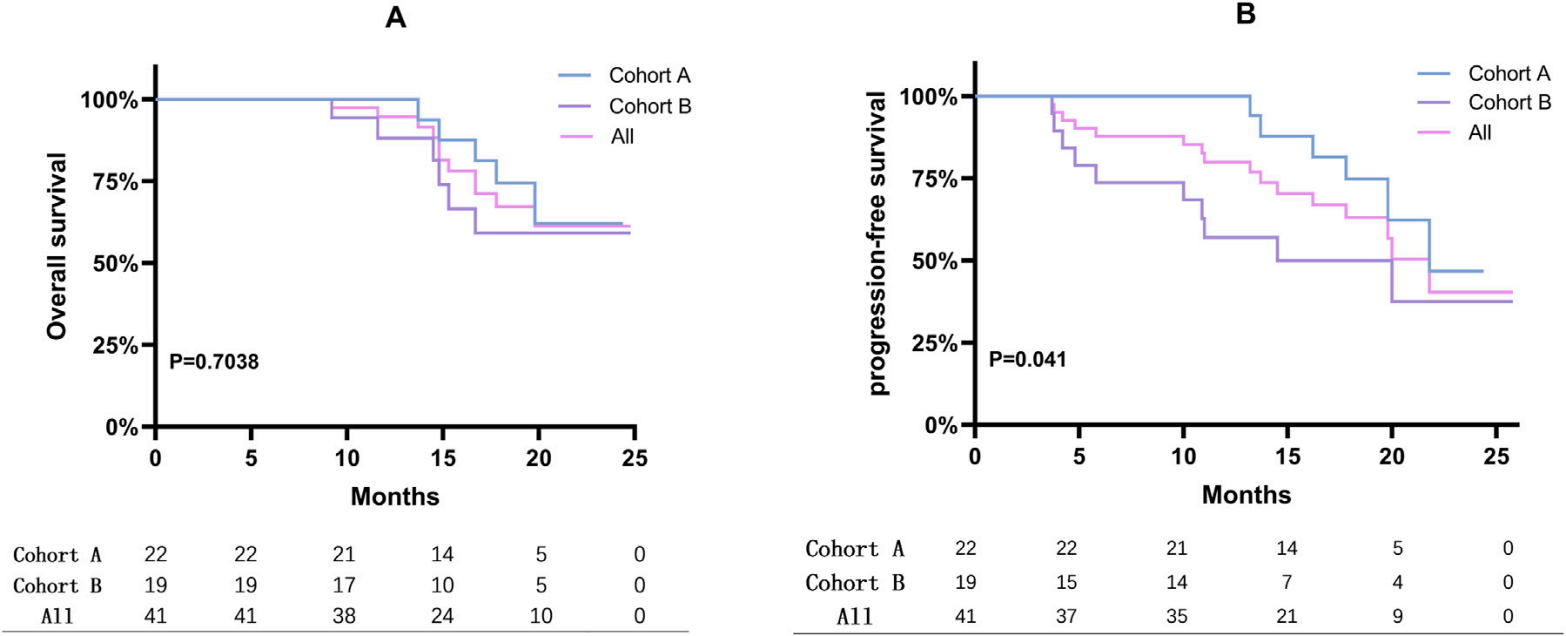
Kaplan-Meier curves displaying PFS and OS. **(A)** Overall survival rates for mCRPC patients were presented using Kaplan-Meier survival curves. **(B)** Kaplan-Meier curves displaying progression-free survival (PFS) for mCRPC patients. The analysis assesses the impact of treatment on disease progression.

After a median follow-up of 16.9 months (range: 3.7–25.8 months), the treatment outcomes and patient prognoses were presented in Figure 1. According to RECIST 1.1 criteria, 2 patients achieved a CR, 18 patients achieved a PR, 5 maintained SD, and 16 experienced PD, as illustrated in Figures 1A, B. Specifically, the median PFS for Cohort A was 21.8 months compared to 14.5 months for Cohort B. The median OS had not yet been reached for either cohort, as shown in Figure 2. Among the evaluated patients, 81.8% in Cohort A and 73.7% in Cohort B achieved a PSA50 response. In terms of tumor size reduction, assessed via imaging, the ORR and DCR for Cohort A were 54.6% and 72.7%, respectively, compared to 42.1% and 47.4% for Cohort B, as detailed in Figures 1D, E.

### Side effects

Following combined treatment with PARP inhibitors and NHT, 85.4% of patients experienced side effects, as detailed in Table 3. The most common AEs included anemia (32/41), fatigue or asthenia (27/41), gastrointestinal symptoms such as nausea and decreased appetite (25/41), and arthralgia (11/41). These AEs were primarily mild to moderate, classified as grade 1 or 2. However, more severe AEs, categorized as grade 3 or 4, occurred in 21 patients, including 9 cases of anemia, 7 of thrombocytopenia, and 9 of neutropenia. These AEs were generally managed through dose discontinuation or reduction, and all affected patients received symptomatic treatment.

**TABLE 3:** Adverse events.

Event	Overall (n = 41)	Cohort A (n = 22)	Cohort B (n = 19)
AEs			
Any	35 (85%)	18 (82%)	17 (89%)
Anemia	32 (78%)	17 (77%)	15 (79%)
Fatigue or asthenia	27 (67%)	9 (43%)	11 (57%)
Decreased appetite	25 (61%)	14 (64%)	11 (57%)
Nausea	23 (56%)	12 (55%)	11 (57%)
Arthralgia	11 (28%)	8 (36%)	3 (14%)
Vomiting	11 (28%)	8 (36%)	3 (14%)
Constipation	11 (28%)	6 (27%)	5 (29%)
Dyspnea	9 (22%)	4 (18%)	5 (29%)
Diarrhea	9 (22%)	6 (27%)	3 (14%)
Back pain	9 (22%)	2 (9%)	8 (43%)
Peripheral edema	2 (6%)	2 (9%)	0 (0%)
Death due to AEs	0	0	0

AEs: adverse events.

### The landscape of HRR mutations in prostate cancer patients

**FIGURE 3:**
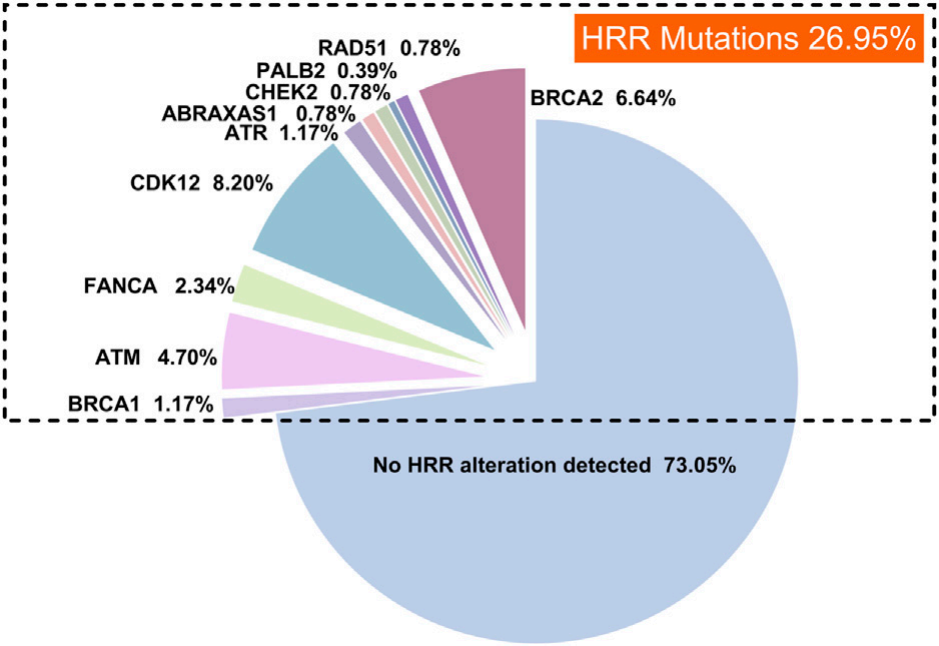
The landscape of HRR mutations in prostate cancer patients.

In this study, we reviewed high-throughput genetic sequencing data from 256 Chinese patients with prostate cancer treated at our institution. Our analysis identified HRR mutations in 26.95% of the patients (69 out of 256). The most common HRR mutations were CDK12 (8.20%, 21/256), BRCA2 (6.64%, 17/256), ATM (4.70%, 12/256), FANCA (2.34%, 6/256), and BRCA1 (1.17%, 3/256), as illustrated in Figure 3.

## Discussions

This study evaluated the combined administration of PARP inhibitors and NHT for efficacy and safety in mCRPC patients harboring HRR gene mutations. This was the first population-based study of PARP inhibitors and NHT’s real-world use and outcomes in mCRPC patients from the Chinese mainland. Across the entire patient cohort, the median PFS was recorded at 21.8 months. Specifically, Cohort A demonstrated a median PFS of 21.8 months, while Cohort B experienced a shorter median PFS of 14.5 months. Efficacy assessments revealed a PSA50 response in 81.8% of patients in Cohort A and 73.7% in Cohort B. ORR was 54.6% in Cohort A and 42.1% in Cohort B, while DCR reached 72.7% and 47.4%, respectively. The toxicity profile was consistent with previous findings (de Bono et al., 2020), with common symptoms including asthenia, anemia, nausea, and anorexia. The majority of adverse events were mild to moderate (grade 1 or 2) in 85.4% of patients, whereas 51.2% experienced severe (grade 3 or 4) adverse events.

The FDA’s approval of olaparib for treating mCRPC marked a significant advancement in precision medicine. The PROfound trial reported a median OS of 17.3 months with olaparib versus 14.0 months for another androgen receptor (AR)-targeted agent, yielding a hazard ratio (HR) of 0.79 (95% confidence interval (CI): 0.61–1.03) (de Bono et al., 2020), highlighting its survival benefits. Pamiparib, developed by BeiGene, Ltd., was a promising investigational inhibitor of PARP1 and PARP2 known for its potent radiosensitizing properties and ability to trap PARP-DNA complexes in preclinical studies (Liu et al., 2023). Our research represented the first evaluation of the real-world effectiveness of PARP inhibitors, including pamiparib, among mCRPC patients in mainland China. In trials like PROpel and TALAPRO-2 involving HRR mutant populations, significant extensions in median PFS were observed with combination therapies compared to controls (Agarwal et al., 2023; Clarke et al., 2022). Specifically, the PROpel trial revealed that the combination of olaparib with abiraterone acetate and prednisone (AAP) led to an rPFS that was not reached, compared to 13.9 months in the placebo plus AAP arm (HR = 0.50; 95% CI: 0.34–0.73). Similarly, the TALAPRO-2 trial reported an rPFS of 27.9 months for the combination of talazoparib with enzalutamide versus 16.4 months for the placebo plus enzalutamide (HR = 0.46; 95% CI: 0.30–0.70). Moreover, the MAGNITUDE trial showed a longer median rPFS in HRR and BRCA1/2 mutant populations treated with niraparib and AAP(26). These trials and our results collectively emphasized the efficacy of combining PARP inhibitors with NHT. Despite variations in specific treatment regimens, our cohort recorded a median PFS of 21.8 months, indicating potential variability in response due to real-world patient heterogeneity and treatment adherence differences. Additionally, significant differences in the PFS Kaplan-Meier curves between cohorts A and B (P = 0.041) supported the findings from the PROfound and TOPARP-B studies (de Bono et al., 2020; Carreira et al., 2021), which showed more significant benefits for patients with BRCA1/2 or ATM mutations treated with PARP inhibitors. Notably, two patients in our cohort achieved radiological CR criteria, although PSA levels were detectable but remained below 0.1, at 0.038 and 0.057, respectively.

Comparing our study’s population with those in RCTs like PROpel reveals important clinical insights (de Bono et al., 2020). While age and ECOG performance status match, differing Gleason scores suggest our cohort includes a broader disease severity spectrum typical in clinical settings, potentially influencing treatment efficacy and safety. Moreover, our real-world setting likely leads to varied treatment adherence and comorbidity management, complicating direct comparisons of therapeutic outcomes and safety.

In this study, PARP inhibitors showed good tolerability among Chinese patients with prostate cancer. The overall incidence of AEs was similar to that observed in a multicenter real-world study in the United States (85.4% vs. 79.1%) (Xie et al., 2024). However, it was lower than the incidence reported in phase III trials (de Bono et al., 2020). This discrepancy in the incidence of severe adverse events between real-world data and clinical trials warrants careful consideration. Our findings suggest potential underreporting of severe adverse events in real-world settings due to less stringent monitoring protocols compared to controlled trial environments. This underreporting could skew perceptions of the tolerability and saety of PARP inhibitors in routine clinical practice. Clinicians should be mindful of this discrepancy when making treatment decisions, and further studies are necessary to develop more rigorous mechanisms for monitoring and reporting adverse events in real-world settings, potentially bridging the gap between clinical trial outcomes and real-world experiences.

Anemia was the most frequent adverse event, consistently occurring across all grades and notably in grade ≥3 events, which had aligned with findings from the TALAPRO-2, PROpel, and MAGNITUDE studies (Agarwal et al., 2023; Clarke et al., 2022; Chi et al., 2023). A critical limitation of current PARP inhibitors was their inability to distinguish effectively between PARP-1 and PARP-2 due to the high homology of their catalytic domains. This nonspecificity was problematic, as PARP-2 is crucial for the survival of progenitor and hematopoietic stem cells, as demonstrated in animal studies. Consequently, inhibiting PARP-2 can lead to significant toxicities, including anemia and neutrophil count fluctuations (Liu et al., 2023). Additionally, our study confirmed that the incidence of grade ≥3 events was consistent with previous reports (Pan et al., 2022), and all side effects were successfully managed with appropriate treatments.

The median follow-up duration in our study was 16.9 months. This timeframe was guided by the typical progression and survival metrics observed in earlier phase studies and trials involving mCRPC treatments (Pan et al., 2022). While this duration allows for an initial assessment of the efficacy of the treatments in terms of PFS and preliminary OS trends, it is acknowledged that these results represent early data. Given that the OS endpoint has not yet been reached, we are committed to continuing the follow-up of our patient cohort. Extended follow-up will enable us to provide a more comprehensive analysis of the long-term efficacy and safety of the treatments. This ongoing surveillance is essential to validate the initial findings and to observe any long-term adverse effects or benefits of the combined therapy regimen.

To enhance our understanding of the full impact of PARP inhibitor therapies and other treatment modalities for mCRPC, future study designs will incorporate a structured approach to QoL assessment. This will involve the inclusion of specific, validated questionnaires that are sensitive to changes in physical, emotional, and social health dimensions, such as the EORTC QLQ-C30 or FACT-P (Kaasa et al., 1995; Diels et al., 2015). These tools are designed to quantitatively measure patient-reported outcomes, ensuring that our findings reflect the nuanced effects of treatments on patients’ quality of life.

Despite the novel insights from our study, several limitations warrant a cautious interpretation of the results. Firstly, as a single-center real-world study with a small patient cohort, random factors may not have been eliminated, and information bias was possible. Secondly, the study lacked a comparative analysis between combination therapies and PARP inhibitors used alone. Thirdly, the brief follow-up period necessitates further research to assess the long-term effects of therapy on survival outcomes and quality of life.

## Conclusion

In conclusion, our study assessed the short-term efficacy and AEs of combined treatment with PARP inhibitors and NHT in patients with mCRPC harboring mutations in HRR genes. The results, observed within a real-world setting, demonstrated effectiveness. The toxicity profiles documented in our study aligned with those from previous clinical trials and were generally tolerable. Furthermore, patients with mutations in BRCA1, BRCA2, or ATM genes exhibited notably greater efficacy compared to those with other HRR gene alterations in the Chinese population.

## Data Availability

The original contributions presented in the study are included in the article/Supplementary Material, further inquiries can be directed to the corresponding author.
