# Supplementary material for: Real-world efficacy and safety of combined first-line treatment with PARP inhibitors and novel hormonal therapy in mCRPC patients with HRR gene mutations
Source: Front Genet. 2024 Dec 6;15:1505163. doi: 10.3389/fgene.2024.1505163 (PMC11659292; doi:10.3389/fgene.2024.1505163)
Supplement: Supplementary file 1 [file DataSheet1.docx]

| **Supplemental Table 1: Patient treatment response and survival data** | | | | | | | | | | | | |
| --- | --- | --- | --- | --- | --- | --- | --- | --- | --- | --- | --- | --- |
| Patient ID | Treatment Regimen | Enrollment Date | Current Medication Cycles | First Follow-up Date and Efficacy Evaluation | Second Follow-up Date and Efficacy Evaluation | Third Follow-up Date and Efficacy Evaluation | Fourth Follow-up Date and Efficacy Evaluation | Fifth Follow-up Date and Efficacy Evaluation | Sixth Follow-up Date and Efficacy Evaluation | Survival Status | Time of Death (months) | Post-Treatment PSA (ng/ml) |
| 1 | Olaparib 300mg bid po + Abiraterone 1000mg qd po | 2022/6/2 | 12 | 2022/10/1 SD | 2023/2/5 PR | 2023/6/17 CR |  |  |  | Alive | - | 0.038 |
| 2 | Olaparib 300mg bid po + Abiraterone 1000mg qd po | 2023/7/5 | 13 | 2023/11/7 SD | 2024/3/15 PD | 2024/8/29 PD |  |  |  | Dead | 2024/9/29 | 0.42 |
| 3 | Olaparib 300mg bid po + Abiraterone 1000mg qd po | 2022/9/1 | 18 | 2023/1/3 SD | 2023/5/16 SD | 2023/9/21 PR | 2024/1/3 PR | 2024/3/22 PR |  | Alive | - | 1.11 |
| 4 | Olaparib 300mg bid po + Abiraterone 1000mg qd po | 2022/3/7 | 24 | 2022/7/1 PD | 2022/11/21 SD | 2023/3/8 SD | 2023/7/21 PR | 2023/11/2 PR |  | Alive | - | 8.1 |
| 5 | Pamiparib 60mg bid po +Abiraterone 1000mg qd po | 2022/3/21 | 19 | 2022/7/30 SD | 2022/11/17 SD | 2023/3/1 SD | 2023/7/21 PD | 2023/11/15 PD |  | Dead | 2023/11/15 | 22.91 |
| 6 | Pamiparib 60mg bid po +Abiraterone 1000mg qd po | 2023/2/14 | 16 | 2023/6/3 SD | 2023/10/21 SD | 2024/2/23 SD | 2024/7/1 SD |  |  | Alive | - | 0.21 |
| 7 | Olaparib 300mg bid po + Abiraterone 1000mg qd po | 2022/11/17 | 21 | 2023/3/21 SD | 2023/7/4 PD | 2023/11/1 SD | 2024/3/19 PR | 2024/9/8 PR |  | Alive | - | 7.77 |
| 8 | Pamiparib 60mg bid po +Abiraterone 1000mg qd po | 2022/9/8 | 20 | 2023/1/13 SD | 2023/5/18 PD | 2023/9/6 SD | 2024/1/17 PD | 2024/6/2 PD |  | Dead | 2024/6/2 | 25.76 |
| 9 | Pamiparib 60mg bid po +Abiraterone 1000mg qd po | 2023/7/23 | 8 | 2023/11/28 PR | 2023/4/5 CR |  |  |  |  | Alive | - | 0.19 |
| 10 | Pamiparib 60mg bid po +Abiraterone 1000mg qd po | 2022/2/25 | 19 | 2022/6/7 PD | 2022/10/3 SD | 2023/2/5 SD | 2023/6/25 SD | 2023/10/10 SD |  | Alive | - | 5.25 |
| 11 | Pamiparib 60mg bid po +Abiraterone 1000mg qd po | 2023/1/27 | 13 | 2023/5/1 SD | 2023/9/21 SD | 2024/3/6 PR |  |  |  | Alive | - | 0.04 |
| 12 | Olaparib 300mg bid po + Abiraterone 1000mg qd po | 2022/5/31 | 16 | 2022/9/21 SD | 2023/1/13 SD | 2023/5/29 PD | 2023/10/6 PD |  |  | Dead | 2023/10/21 | 0.1 |
| 13 | Olaparib 300mg bid po + Abiraterone 1000mg qd po | 2023/5/7 | 12 | 2023/9/18 PR | 2024/1/19 PR | 2024/6/4 PR |  |  |  | Alive | - | 0.28 |
| 14 | Pamiparib 60mg bid po +Abiraterone 1000mg qd po | 2022/3/9 | 17 | 2022/7/21 SD | 2022/11/7 SD | 2023/3/27 SD | 2023/9/3 SD |  |  | Alive | - | 0.83 |
| 15 | Pamiparib 60mg bid po +Enzalutamide 160mg qd po | 2022/3/18 | 25 | 2022/7/8 SD | 2022/11/14 SD | 2023/3/26 PR | 2023/7/21 PR | 2023/11/7 PR | 2024/4/21 PR | Alive | - | 0.96 |
| 16 | Olaparib 300mg bid po + Abiraterone 1000mg qd po | 2023/7/12 | 13 | 2023/11/10 SD | 2024/3/9 PD | 2024/9/3 PD |  |  |  | Dead | 2024/9/3 | 25.2 |
| 17 | Pamiparib 60mg bid po +Abiraterone 1000mg qd po | 2022/7/21 | 10 | 2022/11/2 PR | 2023/3/8 PR | 2023/5/21 PR |  |  |  | Alive | - | 0.11 |
| 18 | Olaparib 300mg bid po + Abiraterone 1000mg qd po | 2022/12/1 | 18 | 2023/4/7 PR | 2023/8/21 PR | 2023/12/4 PR | 2024/6/4 SD |  |  | Alive | - | 0.31 |
| 19 | Olaparib 300mg bid po + Enzalutamide 160mg qd po | 2023/1/4 | 17 | 2023/5/13 SD | 2023/9/29 SD | 2024/1/3 PD | 2024/6/28 PD |  |  | Dead | 2024/6/28 | 17.91 |
| 20 | Pamiparib 60mg bid po +Abiraterone 1000mg qd po | 2022/5/1 | 19 | 2022/9/17 SD | 2023/1/21 SD | 2023/5/7 SD | 2023/9/5 PR | 2023/12/16 PR |  | Alive | - | 0.81 |
| 21 | Olaparib 300mg bid po + Abiraterone 1000mg qd po | 2022/2/2 | 13 | 2022/6/9 SD | 2022/10/24 SD | 2023/3/13 PR |  |  |  | Alive | - | 0.23 |
| 22 | Olaparib 300mg bid po + Abiraterone 1000mg qd po | 2022/3/21 | 21 | 2022/7/13 SD | 2022/11/4 PR | 2023/3/29 SD | 2023/7/23 SD | 2023/12/15 PR |  | Alive | - | Undetectable |
| 23 | Pamiparib 60mg bid po +Abiraterone 1000mg qd po | 2022/7/2 | 20 | 2022/11/18 SD | 2023/3/2 SD | 2023/7/29 PR | 2023/11/27 PR | 2024/3/2 PR |  | Alive | - | 0.47 |
| 24 | Pamiparib 60mg bid po + Enzalutamide 160mg qd po | 2022/9/2 | 10 | 2023/1/19 SD | 2023/5/21 SD | 2023/7/2 PD |  |  |  | Dead | 2023/11/26 | 5.67 |
| 25 | Pamiparib 60mg bid po +Abiraterone 1000mg qd po | 2022/7/29 | 20 | 2022/11/21 PR | 2023/3/4 PR | 2023/7/21 SD | 2023/11/19 PD | 2024/3/8 PD |  | Alive | - | 0.06 |
| 26 | Pamiparib 60mg bid po +Abiraterone 1000mg qd po | 2022/3/21 | 21 | 2022/7/5 SD | 2022/11/19 SD | 2023/3/29 PR | 2023/8/9 PR |  |  | Alive | - | 3.04 |
| 27 | Pamiparib 60mg bid po +Abiraterone 1000mg qd po | 2022/3/26 | 11 | 2022/7/1 SD | 2022/11/7 SD | 2023/2/26 PD |  |  |  | Dead | 2023/3/14 | 6.96 |
| 28 | Pamiparib 60mg bid po +Abiraterone 1000mg qd po | 2023/10/3 | 10 | 2024/2/8 SD | 2024/6/18 PD | 2024/9/1 PD |  |  |  | Alive | - | 0.4 |
| 29 | Pamiparib 60mg bid po + Enzalutamide 160mg qd po | 2022/7/28 | 17 | 2022/11/4 SD | 2023/3/17 SD | 2023/7/28 PR | 2024/1/19 PR |  |  | Alive | - | 0.69 |
| 30 | Pamiparib 60mg bid po +Abiraterone 1000mg qd po | 2024/3/29 | 3 | 2024/7/23 PD |  |  |  |  |  | Alive | - | 16.64 |
| 31 | Olaparib 300mg bid po + Abiraterone 1000mg qd po | 2022/2/4 | 25 | 2022/6/27 SD | 2022/10/15 SD | 2023/2/2 SD | 2023/6/19 PR | 2023/10/21 PR | 2024/3/28 PR | Alive | - | 0.7 |
| 32 | Pamiparib 60mg bid po + Enzalutamide 160mg qd po | 2023/1/13 | 14 | 2023/5/1 SD | 2023/9/12 SD | 2024/1/26 PD | 2024/3/28 PD |  |  | Dead | 2023/10/10 | 2.27 |
| 33 | Olaparib 300mg bid po + Abiraterone 1000mg qd po | 2023/3/2 | 19 | 2023/7/28 PR | 2023/11/25 PR | 2024/3/2 PR | 2024/7/23 PR | 2024/10/3 PR |  | Alive | - | 0.06 |
| 34 | Pamiparib 60mg bid po + Enzalutamide 160mg qd po | 2024/2/7 | 4 | 2024/6/15 PD |  |  |  |  |  | Alive | - | 27.75 |
| 35 | Olaparib 300mg bid po + Abiraterone 1000mg qd po | 2022/11/23 | 16 | 2023/3/2 PR | 2023/7/21 PR | 2023/11/1 PR | 2024/4/20 PR |  |  | Alive | - | 16.07 |
| 36 | Pamiparib 60mg bid po + Enzalutamide 160mg qd po | 2022/5/7 | 5 | 2022/11/1 PD |  |  |  |  |  | Dead | 2023/9/28 | 0.16 |
| 37 | Pamiparib 60mg bid po + Abiraterone 1000mg qd po | 2022/6/28 | 21 | 2022/10/14 SD | 2023/2/17 PR | 2023/6/21 PR | 2023/10/14 PR | 2024/3/1 PR |  | Alive | - | 0.39 |
| 38 | Olaparib 300mg bid po + Abiraterone 1000mg qd po | 2024/1/3 | 3 | 2024/4/24 PD |  |  |  |  |  | Alive | - | 25.9 |
| 39 | Pamiparib 60mg bid po +Abiraterone 1000mg qd po | 2023/4/27 | 14 | 2023/8/16 SD | 2023/12/1 SD | 2024/6/27 PR |  |  |  | Dead | 2024/7/12 | 4.3 |
| 40 | Olaparib 300mg bid po + Abiraterone 1000mg qd po | 2023/8/1 | 11 | 2023/12/19 SD | 2024/4/19 SD | 2024/7/4 SD |  |  |  | Alive | - | 0.11 |
| 41 | Pamiparib 60mg bid po +Abiraterone 1000mg qd po | 2023/2/13 | 4 | 2023/6/21 PD |  |  |  |  |  | Dead | 2024/5/22 | 8.32 |

**Bid: twice a day; qd: once a day; po: by mouth (oral administration);** **Complete Response (CR); Partial Response (PR); Stable Disease (SD); Progressive Disease (PD)**

**Supplemental Table 2: Patient baseline data**

| Patient ID | Cohort | Age | ECOG Score | Baseline PSA | Gleason Score | Metastasis Site | HRR_Mutation |
| --- | --- | --- | --- | --- | --- | --- | --- |
| 1 | A | 78 | 1 | 16.8 | 8 | Lymph nodes | BRCA2 |
| 2 | A | 73 | 1 | 21 | 9 | Bone | FANCA |
| 3 | A | 70 | 1 | 7.4 | 6 | Bone; Lymph nodes | ATM |
| 4 | A | 75 | 0 | 40.5 | 7 | Bone | BRCA2 |
| 5 | A | 72 | 1 | 15.8 | 6 | Bone | CDK12 |
| 6 | A | 78 | 1 | 20.8 | 7 | Bone; Lymph nodes | CDK12 |
| 7 | A | 81 | 1 | 25.9 | 7 | Bone | BRCA2 |
| 8 | A | 82 | 1 | 16.1 | 9 | Bone; Lymph nodes | CHEK2 |
| 9 | A | 70 | 1 | 18.5 | 9 | Lymph nodes | BRCA1 |
| 10 | A | 66 | 0 | 32.8 | 10 | Bone | ATM |
| 11 | A | 72 | 0 | 4.1 | 9 | Bone | ATM |
| 12 | A | 70 | 0 | 9.6 | 8 | Bone | CDK12 |
| 13 | A | 58 | 1 | 5.6 | 9 | Bone ;Liver | BRCA2 |
| 14 | A | 82 | 0 | 41.7 | 9 | Bone; Lymph nodes | ABRAXAS1 |
| 15 | A | 64 | 1 | 9.6 | 7 | Lung | BRCA2 |
| 16 | A | 66 | 0 | 18 | 6 | Bone; Lymph nodes | FANCA |
| 17 | A | 81 | 1 | 11.1 | 7 | Bone; Lymph nodes | CDK12 |
| 18 | A | 58 | 1 | 15.5 | 7 | Bone; Lymph nodes | ATM |
| 19 | A | 69 | 1 | 9 | 6 | Bone; Lung | ATR |
| 20 | A | 65 | 1 | 27 | 7 | Bone; Lung ;Lymph nodes | RAD51B |
| 21 | A | 81 | 0 | 4.5 | 9 | Bone | ABRAXAS1 |
| 22 | A | 68 | 1 | 0.2 | 10 | Bone; Lymph nodes | ATM |
| 23 | B | 79 | 1 | 11.7 | 7 | Bone; Lymph nodes | CHEK2 |
| 24 | B | 77 | 0 | 8.1 | 6 | Bone; Lung ;Lymph nodes | BRCA2 |
| 25 | B | 68 | 1 | 0 | 7 | Bone; Lymph nodes | CDK12 |
| 26 | B | 63 | 1 | 15.2 | 9 | Bone; Lung ;Lymph nodes | BRCA2 |
| 27 | B | 63 | 1 | 4.8 | 8 | Bone | BRCA2 |
| 28 | B | 61 | 1 | 46 | 9 | Bone; Lung | BRCA2 |
| 29 | B | 65 | 1 | 2.3 | 7 | Bone; Lung ;Lymph nodes | CDK12 |
| 30 | B | 70 | 1 | 10.4 | 9 | Bone;Liver | ATM |
| 31 | B | 67 | 0 | 14 | 9 | Bone | CDK12 |
| 32 | B | 69 | 1 | 45.4 | 7 | Lymph nodes | ATM |
| 33 | B | 67 | 0 | 5.9 | 9 | Bone | BRCA2 |
| 34 | B | 69 | 1 | 22.2 | 9 | Lung | CDK12 |
| 35 | B | 68 | 1 | 35.7 | 9 | Bone; Lymph nodes | RAD51C |
| 36 | B | 72 | 1 | 15.5 | 7 | Bone; Lymph nodes | BRCA1 |
| 37 | B | 62 | 0 | 2.6 | 9 | Bone | CDK12 |
| 38 | B | 61 | 1 | 18.5 | 9 | Bone | BRCA2 |
| 39 | B | 76 | 1 | 43 | 9 | Bone; Lymph nodes | ATM |
| 40 | B | 83 | 0 | 10.9 | 9 | Bone | PALB2 |
| 41 | B | 83 | 1 | 41.6 | 9 | Bone | BRCA2 |

**Supplemental Table 3: Patient's Adverse Reaction**

| **Patient ID** | **Cohort** | **Adverse Reaction** |
| --- | --- | --- |
| 1 | A | Anemia; Decreased appetite; Arthralgia; Diarrhea |
| 2 | A | Anemia; Fatigue or asthenia; Decreased appetite; Nausea; Vomiting; Diarrhea |
| 3 | A | Anemia; Fatigue or asthenia; Decreased appetite; Nausea |
| 4 | A | Anemia; Fatigue or asthenia; Nausea; Arthralgia; Diarrhea |
| 5 | A | - |
| 6 | A | Anemia; Decreased appetite; Arthralgia; Vomiting; Dyspnea; Diarrhea |
| 7 | A | Anemia; Decreased appetite; Nausea; Constipation; Dyspnea |
| 8 | A | Anemia; Fatigue or asthenia Nausea; Vomiting; Dyspnea; Diarrhea |
| 9 | A | Anemia; Decreased appetite; Nausea; Vomiting; Constipation |
| 10 | A | Anemia; Fatigue or asthenia; Constipation; Dyspnea; Diarrhea |
| 11 | A | Anemia; Decreased appetite; Arthralgia; Vomiting; Constipation |
| 12 | A | - |
| 13 | A | Decreased appetite; Nausea; Arthralgia; Constipation |
| 14 | A | Anemia; Fatigue or asthenia; Decreased appetite; Constipation |
| 15 | A | Anemia; Decreased appetite; Nausea; Vomiting; Back pain |
| 16 | A | - |
| 17 | A | Anemia; Fatigue or asthenia; Decreased appetite; Back pain |
| 18 | A | Anemia; Decreased appetite; Nausea; Arthralgia; Vomiting |
| 19 | A | Anemia; Fatigue or asthenia; Nausea; Arthralgia; Peripheral edema |
| 20 | A | Anemia; Decreased appetite; Nausea; Arthralgia; Vomiting; Peripheral edema |
| 21 | A | Anemia; Fatigue or asthenia; Decreased appetite; Nausea |
| 22 | A | - |
| 23 | B | Anemia; Decreased appetite; Back pain |
| 24 | B | Anemia; Nausea; Back pain |
| 25 | B | Fatigue or asthenia; Nausea; Vomiting; Back pain |
| 26 | B | - |
| 27 | B | Anemia; Fatigue or asthenia; Nausea; Vomiting; Back pain |
| 28 | B | Fatigue or asthenia; Nausea; Vomiting; Diarrhea |
| 29 | B | Anemia; Fatigue or asthenia; Decreased appetite; Arthralgia |
| 30 | B | Fatigue or asthenia; Nausea; Constipation; Dyspnea; Diarrhea |
| 31 | B | Anemia; Fatigue or asthenia; Nausea; Dyspnea; Diarrhea |
| 32 | B | Anemia; Fatigue or asthenia; Constipation |
| 33 | B | Anemia; Fatigue or asthenia; Nausea; Constipation |
| 34 | B | Fatigue or asthenia; Decreased appetite; Dyspnea |
| 35 | B | Anemia; Decreased appetite; Nausea; Dyspnea; Back pain |
| 36 | B | - |
| 37 | B | Anemia; Decreased appetite; Dyspnea; Back pain |
| 38 | B | Anemia; Fatigue or asthenia; Arthralgia |
| 39 | B | Anemia; Decreased appetite; Constipation; Back pain |
| 40 | B | Anemia; Decreased appetite; Arthralgia |
| 41 | B | Anemia; Decreased appetite; Constipation; Back pain |
